# Supplementary material for: The A Body Shape Index Might Be a Stronger Predictor of Chronic Kidney Disease Than BMI in a Senior Population
Source: Int J Environ Res Public Health. 2021 Dec 7;18(24):12874. doi: 10.3390/ijerph182412874 (PMC8701813; doi:10.3390/ijerph182412874)
Supplement: Supplementary file 1 [file ijerph-18-12874-s001.zip › ijerph-1452453-supplementary.pdf]

The A Body Shape Index might be a stronger predictor of chronic kidney disease than BMI in a senior population

Bokun Kim, Gwon-min Kim, Eonho Kim, Jong-Hwan Park, Tomonori Isobe, Takeji Sakae, Sechang Oh

Table of contents

Supplementary methods

Supplementary results

Table S1

Table S2

Supplementary methods

Table S1

Descriptive statistics and comparisons between male and female participants were performed.

Table S2

Group classification

eGFR was defined according to the new Japanese coefficient-modified Modification of Diet in Renal Disease study equation: eGFR (mL/min/1.73 m^2) = 194 × (serum creatinine)$^{-1.094}$ × (age)$^{-0.287}$ (× 0.739 for women) [15, 21, 22]. The participants were separated into three groups based on eGFR tertiles: the normal (≥60.0 mL/min/1.73 m^2) group, mild CKD (45.0–59.9 mL/min/1.73 m^2) group, and moderate-to-severe CKD (<45.0 mL/min/1.73 m^2) group [15, 21, 22].

Blood pressure

Blood pressure was assessed three times in the right arm using an electronic sphygmomanometer, and average systolic (SBP) and diastolic (DBP) blood pressure values were computed.

Biochemical assessments

Blood samples were collected in the morning following an ≥ 8 h fast. Levels of total cholesterol (TC) and triglycerides (TG), high-density lipoprotein cholesterol (HDL-C), fasting plasma glucose (FPG), and HbA1C were analyzed using an enzymatic method, homogeneous enzymatic colorimetric method, hexokinase UV method, and high-performance liquid chromatography, respectively, at a certified laboratory (Seegene Medical Foundation, Seoul, Korea).

Covariates

Information that is known or suspected to be associated with chronic kidney disease was adopted as potential confounding factors: Nutrition, alcohol consumption, education level, handgrip strength, household income, moderate to vigorous physical activity (MVPA), sex, and smoking. Nutrition data were collected in a single 24-h recall method, and total energy and each dietary intakes such as carbo, protein and fat were calculated. Self-reported alcohol consumption was classified as never, ≤ once/week, 2–3 times/week, or ≥ 4 times/week; education level as primary, middle and high school, or ≥ college; and smoking habit as never, former, or current smoking. Household income was classified into quartiles. To evaluate muscular

strength, handgrip strength was assessed three times for both the left and right hands, and average values were computed. MVPA was evaluated using the International Physical Activity Questionnaire.

Statistical analysis

Data are presented as mean ± standard deviation, or number of cases (%). An independent t-test or the Mann–Whitney U test were adopted to compare parameters between male and female patients. One-way analysis of variance (ANOVA) was used to compare mean blood pressure, biochemical parameters, physical activity, handgrip strength, and nutrition among the three groups. The Bonferroni post-hoc test was used when ANOVA showed significant differences ($P < 0.05$). In case of abnormal data distribution, the Mann–Whitney U test was used to analyze differences between the groups ($P < 0.05$). Statistical analyses were performed using SPSS software, version 20.0 (IBM, Inc., Armonk, NY, USA).

Supplementary results

Table S1

For overall, male, and female participants, mean MVPA was 79.9 (*SD*, 209.8), 91.4 (*SD*, 246.9), and 70.0 (*SD*, 171.1), respectively, and mean protein intake was 68.8 (*SD*, 38.2), 69.9 (*SD*, 40.0), and 67.8 (*SD*, 36.5), respectively. The values of these two parameters were significantly higher in men than in women ($P < 0.05$ for both). Significant differences between the sexes were observed in household income, education level, alcohol consumption, and smoking ($P < 0.001$). For other parameters, no sex differences were observed.

Table S2

Overall, the trend test revealed a significant increasing tendency in SBP, glucose, HbA1c, and triglycerides across the groups from *N* to S_{CKD} (*SS* = 12.53, 10.00, 13.35, and 6.02, respectively; $P < 0.001$). The opposite tendency was observed for HDL-C, total energy, and protein and fat intake (*SS* = −6.06, −5.91, −8.10, and −11.52, respectively; $P < 0.001$). No significant tendency was observed for DBP, cholesterol, MVPA, handgrip strength, or carbohydrate intake. The post-hoc test demonstrated significant differences among the three groups for all parameters, except for DBP and MVPA. The *N*, M_{CKD}, and S_{CKD} groups ranked in descending order for total energy,

and protein and fat intake, but in ascending order for SBP. Glucose and HbA1c, and HDL-C values in the M_{CKD} and S_{CKD} groups did not differ, although the values were significantly higher or lower, respectively, compared with those of the *N* group. Cholesterol and carbohydrate intake values in the *N* group were lower than those of the M_{CKD} group. Triglyceride and handgrip strength values in the *N* group were lower and higher, respectively, than those of the S_{CKD} group.

In male participants, the trend test revealed a significant decreasing tendency across the groups from *N* to S_{CKD} in HDL-C ($SS = -3.93$, $P < 0.001$), total energy intake ($SS = -3.84$, $P < 0.05$), protein intake ($\mathrm{SS} = -5.35$, $P < 0.001$), and fat intake ($SS = -7.38$, $P < 0.001$). The opposite tendency was observed for SBP, glucose, HbA1c, and triglycerides (SS = 8.38, 7.72, 10.37, and 4.10, respectively; $P < 0.001$). No significant tendency was observed for DBP, cholesterol, MVPA, handgrip strength, or carbohydrate intake. The post-hoc test demonstrated significant differences among the three groups for all parameters, except for DBP, cholesterol, triglycerides, MVPA, handgrip strength, and carbohydrate intake. The *N*, M_{CKD}, and S_{CKD} groups ranked in descending order for protein and fat intake, but in ascending order for SBP and HbA1c. Glucose values in the M_{CKD} and S_{CKD} groups did not differ, although the values in both groups were significantly higher than those of the *N* group. HDL-C and total energy intake values in the *N* and M_{CKD} groups did not differ, although the values in both groups were significantly higher than those of the S_{CKD} group.

In female participants, the trend test revealed a significant decreasing tendency across the groups from *N* to S_{CKD} in protein ($SS = -6.12$, $P < 0.001$) and fat intake ($SS = -8.88$, $P < 0.001$). The opposite tendency was observed for SBP ($SS = 9.32$, $P < 0.001$), glucose ($SS = 6.46$, $P < 0.001$), HbA1c ($SS = 8.60$, $P < 0.001$), and triglyceride ($SS = 3.40$, $P < 0.01$). However, no significant tendency was observed for DBP, cholesterol, HDL-C, MVPA, handgrip strength, total energy intake, or carbohydrate intake. The post-hoc test revealed significant differences among the three groups in all parameters, except for DBP, cholesterol, triglycerides, MVPA, handgrip strength, and carbohydrate intake. The *N*, M_{CKD}, and S_{CKD} groups ranked in descending order for protein and fat intake. SBP, HbA1c, HDL-C, and total energy intake values in the M_{CKD} and S_{CKD} groups did not differ. However, SBP and HbA1c, and HDL-C and total energy intake values, in the M_{CKD} and S_{CKD} groups, were significantly higher and lower, respectively, than those of the *N* group.

Table S1. Characteristics of study subjects

	Overall (n = 7053)	Male (n = 3257)	Female (n = 3796)	*P* Value
SBP, mm Hg	120.6 ± 17.0	120.5 ± 17.0	120.8 ± 16.9	= 0.510
DBP, mm Hg	75.7 ± 10.1	75.7 ± 10.1	75.6 ± 10.1	= 0.667
Glucose, mg/dL	101.9 ± 23.9	101.4 ± 22.8	102.3 ± 24.8	= 0.102
HbA1c, %	5.8 ± 0.8	5.8 ± 0.8	5.8 ± 0.8	= 0.431
TC, mg/dL	192.7 ± 37.4	193.2 ± 37.3	192.3 ± 37.5	= 0.334
HDLC, mg/dL†	50.5 ± 12.6	50.6 ± 12.5	50.5 ± 12.8	= 0.666
TG, mg/dL	135.5 ± 98.3	134.8 ± 94.5	136.0 ± 101.4	= 0.629
Handgrip strength, kg	28.3 ± 9.7	28.4 ± 9.8	28.2 ± 9.7	= 0.318
MVPA, min†	79.9 ± 209.8	91.4 ± 246.9	70.0 ± 171.1	< 0.05
TEI, kcal/d	1966.2 ± 870.9	1980.9 ± 874.3	1953.6 ± 867.8	= 0.190
Carbohydrate, g	308.8 ± 130.8	310.0 ± 130.8	307.8 ± 130.9	= 0.477
Protein, g	68.8 ± 38.2	69.9 ± 40.0	67.8 ± 36.5	< 0.05
Fat, g	40.7 ± 31.9	41.4 ± 33.0	40.0 ± 31.0	= 0.068
Household income (%)				
Low	2632 (37.3)	1038 (31.9)	1594 (42.0)	< 0.001
Lower-middle	2028 (28.8)	989 (30.4)	1039 (27.4)	
Upper middle	1330 (18.9)	671 (20.6)	659 (17.4)	
High	1063 (15.1)	559 (17.2)	504 (13.3)	
Education level (%)				
Primary school	3556 (50.4)	1156 (35.5)	2400 (63.2)	< 0.001
Middle school	1179 (16.7)	600 (18.4)	579 (15.3)	
High school	1432 (20.3)	878 (27.0)	554 (14.6)	
College	886 (12.6)	623 (19.1)	263 (6.9)	
Alcohol consumption (%)				
Never	3052 (43.3)	901 (27.7)	2151 (56.7)	< 0.001
≤ once a week	2653 (37.6)	1203 (36.9)	1450 (38.2)	
2-3 times/week	747 (10.6)	617 (18.9)	130 (3.4)	
≥ 4 times/week	601 (8.5)	536 (16.5)	65 (1.7)	
Smoking (%)				
Never	4228 (59.9)	643 (19.7)	3585 (94.4)	< 0.001
Former smoking	2046 (29.0)	1923 (59.0)	123 (3.2)	
Current smoking	779 (11.0)	691 (21.2)	88 (2.3)	

Values are means ± SD. SBP = Systolic blood pressure; DBP = Diastolic blood pressure; Total cholesterol; HDLC = High density lipoprotein cholesterol; TG = Triglyceride; MVPA = Moderate to vigorous physical activity; HS = Handgrip strength; TEI = Total energy intake

Table S2. Overall and gender-specific differences and trends of subjects by eGFR category

	eGFR category (mL/min/1.73 m²)					
	N<br>eGFR ≥ 60<br>(95% CI)	*M*CKD<br>eGFR 45-59.9<br>(95% CI)	*S*CKD<br>eGFR < 45<br>(95% CI)	*P* for difference	*SS*	*P* for trend‡
**Overall**	**n = 3800**	**n = 2595**	**n = 658**			
SBP, mmHg†	118.4 ± 16.2<br>(117.9, 118.9)	122.6 ± 17.1<br>(121.9, 123.2)	125.8 ± 18.4<br>(124.4, 127.2)	A < B < C	12.53	< 0.001
DBP, mmHg	75.4 ± 9.9<br>(75.1, 75.8)	75.9 ± 10.1<br>(75.5, 76.3)	76.0 ± 10.9<br>(75.1, 76.8)	NS	1.84	= 0.065
Glucose, mg/dL	100.5 ± 23.5<br>(99.8, 101.3)	103.3 ± 24.0<br>(102.4, 104.2)	104.1 ± 25.4<br>(102.2, 106.1)	A < B, C	10.00	< 0.001
HbA1c, %	5.7 ± 0.8<br>(5.7, 5.7)	5.8 ± 0.8<br>(5.8, 5.9)	5.9 ± 0.8<br>(5.9, 6.0)	A < B, C	13.35	< 0.001
TC, mg/dL†	192.0 ± 36.0<br>(190.8, 193.1)	193.9 ± 38.6<br>(192.4, 195.3)	192.3 ± 40.3<br>(189.2, 195.4)	A < B	1.57	= 0.118
HDLC, mg/dL	51.2 ± 12.6<br>(50.8, 51.6)	49.9 ± 12.7<br>(49.4, 50.4)	48.8 ± 12.3<br>(47.9, 49.8)	A > B, C	-6.06	< 0.001
TG,mg/dL	133.1 ± 103.1<br>(129.8, 136.3)	136.9 ± 92.2<br>(133.4, 140.5)	143.6 ± 92.2<br>(136.5, 150.6)	A < C	6.02	< 0.001
MVPA, min/wk†	85.0 ± 229.9<br>(77.7, 92.4)	74.2 ± 183.8<br>(67.2, 81.3)	72.2 ± 181.8<br>(58.3, 86.1)	NS	-0.70	= 0.482
Handgrip strength, kg	28.4 ± 9.6<br>(28.1, 28.7)	28.3 ± 10.0<br>(27.9, 28.7)	27.4 ± 9.6<br>(26.7, 28.1)	A > C	-1.89	= 0.059
TEI, kcal/d†	2019.2 ± 910.1<br>(1990.3, 2048.2)	1930.1 ± 828.8<br>(1898.2, 1962.0)	1802.4 ± 768.1<br>(1743.6, 1861.2)	A > B > C	-5.91	< 0.001
Carbo, g†	307.8 ± 133.9<br>(303.6, 312.1)	312.2 ± 129.5<br>(307.2, 317.2)	301.3 ± 117.4<br>(292.3, 310.3)	A < B	0.96	= 0.336
Protein, g†	71.7 ± 39.8<br>(70.4, 73.0)	66.5 ± 36.0<br>(65.1, 67.8)	61.0 ± 35.5<br>(58.3, 63.8)	A > B > C	-8.10	< 0.001
Fat, g†	44.1 ± 33.9<br>(43.0, 45.2)	37.7 ± 29.1<br>(36.6, 38.8)	32.9 ± 28.0<br>(30.8, 35.0)	A > B > C	-11.52	< 0.001
**Male**	**n = 1752**	**n = 1185**	**n = 320**			
SBP, mmHg†	118.3 ± 16.1<br>(117.5, 119.1)	122.2 ± 16.9<br>(121.2, 123.1)	126.3 ± 19.7<br>(124.2, 128.5)	A < B < C	8.38	< 0.001
DBP, mmHg	75.5 ± 9.9<br>(75.1, 76.0)	76.0 ± 10.1<br>(75.4, 76.6)	75.8 ± 10.9<br>(74.5, 77.0)	NS	0.88	= 0.377
Glucose, mg/dL	100.0 ± 22.0<br>(98.9, 101.0)	102.6 ± 22.9<br>(101.3, 103.9)	104.7 ± 25.9<br>(101.9, 107.5)	A < B, C	7.72	< 0.001
HbA1c, %	5.7 ± 0.8<br>(5.7, 5.7)	5.8 ± 0.8<br>(5.8, 5.9)	6.0 ± 0.8<br>(5.8, 6.0)	A < B < C	10.37	< 0.001
TC, mg/dL	191.2 ± 36.5<br>(190.4, 193.8)	195.2 ± 38.1<br>(193.0, 197.3)	191.7 ± 38.5<br>(187.5, 196.0)	NS	1.82	= 0.069
HDLC, mg/dL	51.2 ± 12.5<br>(50.6, 51.8)	50.2 ± 12.7<br>(49.5, 50.9)	48.3 ± 11.3<br>(47.1, 49.6)	A, B > C	-3.93	< 0.001
TG, mg/dL	132.2 ± 100.3	137.3 ± 88.4	140.4 ± 82.6	NS	4.10	< 0.001

	(127.5, 136.9)	(132.3, 142.3)	(131.3, 149.4)			
MVPA, min/wk†	96.2 ± 269.9 (83.5, 108.8)	88.5 ± 223.1 (75.8, 101.2)	76.0 ± 193.3 (54.8, 97.3)	NS	0.34	= 0.737
Handgrip strength, kg	28.2 ± 9.7 (27.8, 28.7)	28.9 ± 9.9 (28.3, 29.4)	27.7 ± 9.6 (26.7, 28.8)	NS	0.81	= 0.416
TEI, kcal/d†	2038.8 ± 914.1 (1984.3, 2070.6)	1972.1 ± 828.2 (1916.9, 2010.7)	1745.2 ± 818.6 (1704.5, 1873.8)	A, B > C	-3.84	< 0.05
Carbo, g	308.5 ± 135.5 (302.2, 314.9)	315.2 ± 127.6 (308.0, 322.5)	298.7 ± 115.2 (286.0, 311.3)	NS	0.80	= 0.427
Protein, g†	72.7 ± 42.2 (70.7, 74.6)	68.5 ± 37.4 (66.3, 70.6)	60.4 ± 34.9 (56.6, 64.3)	A > B > C	-5.35	< 0.001
Fat, g†	44.4 ± 34.7 (42.8, 46.0)	39.1 ± 30.3 (37.3, 40.8)	33.8 ± 31.1 (30.4, 37.2)	A > B > C	-7.38	< 0.001
**Female**	**n = 2048**	**n = 1410**	**n = 338**			
SBP, mmHg	118.5 ± 16.3 (117.8, 119.2)	122.9 ± 17.2 (122.0, 123.8)	125.3 ± 17.1 (123.5, 127.1)	A < B, C	9.32	< 0.001
DBP, mmHg	75.4 ± 9.9 (74.9, 75.8)	75.9 ± 10.2 (75.3, 76.4)	76.1 ± 10.9 (75.0, 77.3)	NS	1.70	= 0.089
Glucose, mg/dL	101.0 ± 24.7 (100.0, 102.1)	103.9 ± 24.9 (102.6, 105.2)	103.6 ± 25.0 (100.9, 106.3)	A < B	6.46	< 0.001
HbA1c, %	5.7 ± 0.8 (5.7, 5.8)	5.8 ± 0.8 (5.8, 5.9)	5.9 ± 0.8 (5.8, 6.0)	A < B, C	8.60	< 0.001
TC, mg/dL†	191.9 ± 35.5 (190.4, 193.4)	192.8 ± 39.0 (190.7, 194.8)	192.9 ± 42.0 (188.4, 197.4)	NS	0.43	= 0.669
HDLC, mg/dL	51.3 ± 12.7 (50.7, 51.8)	49.7 ± 12.7 (49.0, 50.3)	49.3 ± 31.1 (47.9, 50.7)	A > B, C	-0.29	= 0.770
TG, mg/dL	133.8 ± 105.4 (129.2, 138.4)	136.6 ± 95.4 (131.6, 141.6)	146.6 ± 100.5 (135.8, 157.3)	NS	3.43	< 0.01
MVPA, min/wk†	75.5 ± 188.8 (67.3, 83.7)	62.2 ± 141.7 (54.8, 69.6)	68.6 ± 170.3 (50.4, 86.8)	NS	-0.27	= 0.784
Handgrip strength, kg	28.6 ± 9.5 (28.2, 29.9)	27.8 ± 10.0 (27.3, 28.3)	27.1 ± 9.5 (26.1, 28.1)	NS	-1.27	= 0.205
TEI, kcal/d†	2012.2 ± 901.5 (1973.1, 2051.3)	1901.8 ± 833.3 (1858.3, 1945.4)	1815.0 ± 768.0 (1732.8, 1897.2)	A > B, C	-1.81	= 0.071
Carbo, g	307.2 ± 132.6 (301.5, 312.9)	309.6 ± 131.0 (302.8, 316.4)	303.8 ± 119.6 (291.0, 316.6)	NS	-0.44	= 0.662
Protein, g†	70.9 ± 37.6 (69.3, 72.5)	64.8 ± 34.6 (63.0, 66.6)	61.6 ± 36.0 (57.8 65.5)	A > B > C	-6.12	< 0.001
Fat, g†	43.8 ± 33.3 (42.4, 45.2)	36.5 ± 28.0 (35.0, 38.0)	32.0 ± 24.7 (29.4, 34.7)	A > B > C	-8.88	< 0.001

Values are means ± SD. †Mann-Whitney U test was applied to assess the difference betwee n groups. ‡Jonckheere-Terpstra test was used to assess the trend among three groups. SS = standardized statistic; NS = not significant, SBP = Systolic blood pressure; DBP = Diastolic blood pressure; Total cholesterol; HDLC = High density lipoprotein cholesterol; TG = Trigly ceride; MVPA = Moderate to vigorous physical activity; TEI = Total energy intake
